# Rabies Control: Could Innovative Financing Break the Deadlock?

**DOI:** 10.3389/fvets.2017.00032

**Published:** 2017-03-09

**Authors:** Susan C. Welburn, Paul G. Coleman, Jakob Zinsstag

**Affiliations:** ^1^Division of Infection and Pathway Medicine, Edinburgh Medical School: Biomedical Sciences, College of Medicine and Veterinary Medicine, The University of Edinburgh, Edinburgh, UK; ^2^Faculty of Infectious and Tropical Diseases, Department of Disease Control, School of Hygiene & Tropical Medicine, London, UK; ^3^H2O Venture Partners, Oxford, UK; ^4^Department of Epidemiology and Public Health, Swiss Tropical and Public Health Institute, Basel, Switzerland; ^5^University of Basel, Basel, Switzerland

**Keywords:** rabies, development impact bonds, zoonotic, neglected tropical diseases, disease control, finance

## Abstract

The neglected zoonotic diseases (NZDs) have been all but eradicated in wealthier countries but remain major causes of ill-health and mortality in over 80 countries across Africa, Asia, and Latin America. The nature of neglect for the NZDs has been ascribed, in part, to underreporting resulting in an underestimation of their global burden that, together with a lack of advocacy, downgrades their relevance to policy-makers and funding agencies. While this may be the case for many NZDs, for rabies this is not the case. The global burden estimates for rabies (931,600 DALYs) more than justify prioritizing rabies control building on the strong advocacy platforms, functioning at local, regional, and global levels (including the Global Alliance for Rabies Control), and commitments from WHO, OIE, and FAO. Simple effective tools for rabies control exist together with blueprints for operationalizing control, yet, despite elimination targets being set, no global affirmative action has been taken. Rabies control demands activities both in the short term and over a long period of time to achieve the desired cumulative gains. Despite the availability of effective vaccines and messaging tools, rabies will not be sustainably controlled in the near future without long-term financial commitment, particularly as disease incidence decreases and other health priorities take hold. While rabies control is usually perceived as a public good, public private partnerships could prove equally effective in addressing endemic rabies through harnessing social investment and demonstrating the cost-effectiveness of control. It is acknowledged that greater attention to navigating local realities in planning and implementation is essential to ensuring that rabies, and other neglected diseases, are controlled sustainably. In the shadows of resource and institutional limitations in the veterinary sector in low- and middle-income countries, sufficient funding is required so that top-down interventions for rabies can more explicitly engage with local project organization capacity and affected communities in the long term. Development Impact Bonds have the potential to secure the financing required to deliver effective rabies control.

## Introduction

More than a decade of advocacy has resulted in ambitious control and elimination targets for neglected tropical diseases (NTDs) set by WHO for 2020. Partnerships have been formed to raise funds and provide advocacy for NTD control, including the Global Programme to Eliminate Lymphatic Filariasis^1^ and the Global Network for NTDs.^2^ Advocacy resulted in the 2012 London Declaration^3^ and WHO Roadmap to accelerate the work to overcome the global impact of 17 NTDs,^4^ followed by the World Health Assembly (WHA) Resolution WHA66.12 on NTDS in May 2013. However, for the neglected zoonotic diseases (NZDs) that were included in this Roadmap (rabies, echinococcosis hydatid disease, leishmaniasis, *Trypanosoma brucei rhodesiense* sleeping sickness, and *Taenia solium* cysticercosis), little progress has been made. Anthrax, brucellosis, and bovine TB were not included in the resolution.

Rabies is one of the most feared human diseases, estimated to cause some 55,000 deaths each year, predominately among children and the rural poor in Asia and Africa (1–3). The rabies virus has a simple route of transmission; *via* saliva from the bite of an infected animal, the rabies virus invades the central nervous system and, in the absence of postexposure prophylaxis (PEP), is fatal once clinical signs appear (4). Symptoms in dogs can be non-specific but often include “hydrophobia,” hypersalivation, respiratory difficulties, biting, and aggression. Since the vast majority of human rabies cases are caused by domestic dogs (5) and an effective vaccine is available, dog vaccination is the most effective control strategy together with dog population management, movement regulations, and the promotion of responsible dog ownership (5–7). A number of initiatives have been undertaken (8–12), and a combination of intensive canine vaccination and surveillance efforts, implemented since the 1980s in Latin America, has shown dramatic progress (13).

Eliminating infection from dogs reduces the demand for costly PEP, although the relationship is not always as predicted and may vary considerably (14, 15). Despite all the evidence of the benefits of targeting the domestic canine reservoir, dog vaccination remains under-prioritized in most low- and middle-income countries (LMIC) with competing health issues and limited resources. Despite a number of successful initiatives having been implemented, erroneous perceptions of operational constraints among policy-makers (lack of knowledge about the dog population, inadequate resources, and wildlife transmission) are barriers to vaccination (5).

To successfully eliminate rabies, vaccination must reach at least 70% of a dog population over consecutive years, yet, despite the feasibility of elimination, programs in Africa struggle with reaching high levels of coverage (16); vaccination rates lower than 30% are considered a “waste of resources” (5). Despite good quality vaccines for dogs, a genuine science of rabies elimination is needed (17) to understand complex social–ecological determinants of vaccination effectiveness (18). Vaccination coverage declines rapidly in dog populations with high turnover rates (19). Most dogs in Africa are owned by a family but are free roaming and generally young; often half of dogs are less than 1 year of age (20–23). Dog bite data, used to infer numbers of human deaths, were used to calculate the threshold density for rabies persistence as 4.5 dogs/km^2^ (1).

Validated estimates of dog populations are essential for planning successful mass dog vaccinations yet in most cases are lacking (12); for example, a study in Tanzania showed that the dog population was six times larger than the official estimate (23). Interventions are influenced by local dog ownership practices; attitudes toward dogs; the ability and willingness of owners to handle their dogs; the location of vaccination points, and the extent of information dissemination and knowledge of rabies, all of which influence compliance (8, 22, 24, 25). Despite higher costs, house-to-house strategies were necessary to achieve 70% coverage in pastoralist communities in Northern Tanzania (26). Capacity and working norms of implementing organizations are also key; most campaigns are planned nationally and delivered at district and subdistrict level. In many African LMIC, a legacy of structural adjustment in the veterinary sector had resulted in reduced capacity in the animal health sector. Large remote geographical areas together with low salaries, insufficient resources, and rigid bureaucratic norms can further inhibit such campaigns, which depend, to a large degree, on adapting strategies to fit community needs. However, even with cheap and effective dog vaccines available and with burdens and costs well understood, there is no guarantee that elimination will be easily achieved (27).

From a human health perspective, a dog bite wound requires cleaning and a postexposure treatment (PET) vaccination is essential, but expensive. As dog-to-dog transmission drives rabies epidemics, PET alone will not eliminate rabies. From an animal health perspective, rabies in cattle, and not dogs, is considered more important, because of the greater economic value of a cow relative to a dog, so national rabies vaccination programs are not prioritized.

## The Problem with Rabies Control

Neglected zoonotic diseases may be described as the neglected NTDs, beset by problems of underreporting that tends to underestimate their global burden and so diminishes their relevance to policy-makers and funding agencies. Interventions in the animal reservoir for NZDs (mass vaccination, drug treatment, and education) must be supported and operationalized across health and agriculture ministries. Long-term national and regional plans for elimination demand significant buy-in from both human and animal health sectors. When a full cross-sector analysis is undertaken and all stake-holder benefits (monetary/non-monetary) are taken into account, interventions for NZDs can become highly cost-effective, and among all neglected NZDs, dog rabies elimination is the lowest hanging fruit, with all the necessary tools for elimination already available (28, 29). However, for rabies, the cost benefits of vaccinating dogs may take many years to be realized and requires universal high coverage to be achieved annually. For example, mathematical models of rabies control in Ndjamena, Chad, suggest the cumulative cost of dog rabies mass vaccination and human PET was equal to the cumulative cost of PET alone after 6 years and only became more cost-effective after 7 years (15). Costs of rabies control are borne almost entirely by people in the developing world where >99% of all fatalities occur and dog owners have not been willing to pay the full costs of vaccination, indicating that rabies control should be considered a public good (30).

There are proven systems to identify those individuals exposed to the rabies virus, most often following a rabid dog bite, and ensure PET is administered promptly to avoid death from rabies, which is otherwise inevitable once the victim starts to display clinical symptoms. Any death due to dog-mediated rabies is a failure of the public and veterinary health systems, but the main constraint to widespread implementation is finance. Poor countries do not have access to the funds required to develop and deliver an appropriate control strategy tailored to their epidemiological conditions that can be implemented over sufficient time to unlock and sustain public health and economic benefits.

## Development Impact Bonds (DIBs)—A New Approach to Funding Rabies Control

The tools for effective control and the evidence that they work have been around for a long time; the constraining factor has been the financing to implement sustained efforts at scale. Traditional financing streams for NTD control in resource poor settings, particularly grant funding through international governmental donors, charitable organizations, or private institutions, have not been available at the levels required to combat the continued burden of rabies. The failure to secure the necessary financing is in part due to the inability to compete against other pressing infectious disease burdens, which have historically secured the majority of the resources going into NTD control. What can be done to break this deadlock and mobilize additional resources and unlock the benefits of achievable rabies control?

**Figure 1:**
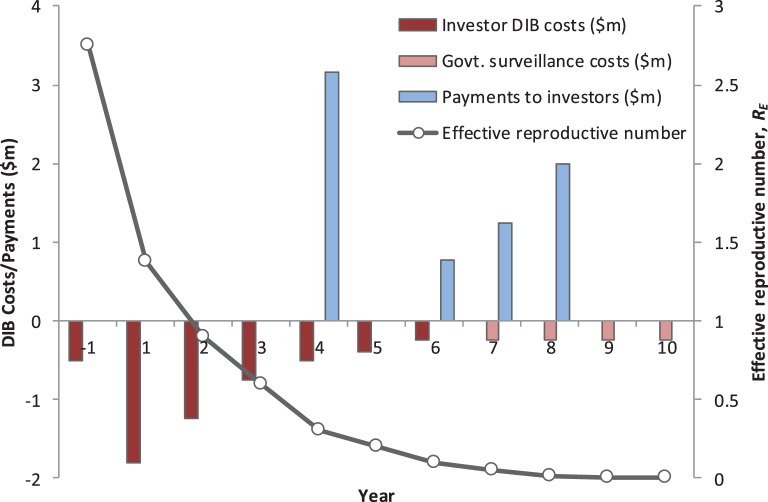
**The annual cash-flow requirements, the performance-related payments to investors, and impact on rabies transmission dynamics for a hypothetical Development Impact Bond (DIB)**. The total cost of the DIB is US$ 6.45 million, including Year 1 establishment costs, with US$ 5.45 million financed by investors (Years 1–6) and US$ 1 million by the Government (Years 7–10 in the post-elimination maintenance phase). This excludes the ongoing government spend providing the routine rabies surveillance platform independent of any specific strengthening or refinements needed to deliver the DIB. The model assumes mass vaccination of 70% of dogs in year 1 (US$ 1.8 million) and then a second round of 70% vaccination spread over 3 years (total cost $2.5 million). Costs for community messaging are included throughout the program. Based on achieving the vaccination targets, the investors receive a payment at the end of Year 4 equal to 66% of the DIB spend over the first 5 years. The remaining payments to the investors in Years 6–8 are linked to reduced rabies transmission and are back-loaded to incentivize a successful transition to embedding the maintenance phase under government spend. The surveillance system is embedded into national veterinary and public health services and assumes an annual cost of US$ 250,000, which includes provision for reactive ring vaccination following confirmed sporadic canine rabies cases and continued use of postexposure prophylaxis following confirmed exposure. The total return to investors is US$ 7.2 million, representing an internal rate of return of 8%.

A new model of sustainable investment in rabies control is required, and DIBs is one approach that potentially could secure the financing required to deliver effective rabies control. We argue that financing structure of a DIB is particularly well suited to financing rabies control and so provides a highly compelling case to donors interested in controlling NTDs in a highly cost-effective manner (Figure 1).

Development Impact Bonds are a form of Social Impact Bonds (SIBs), which are themselves a form of payment for results (31). SIBs have been applied to address a variety of societal problems primarily across the developed world, and although the number and size of transaction is small, the market is growing rapidly (31). In developing settings, DIBs, while far from being a panacea, have been advocated as potentially important in helping address a broad range of inequalities including improved public health provision (32), childhood development (33), and infectious disease control (34).

More broadly, DIBs are one example of a new form of social impact financing in which donor or government payments are structured around the delivery of specific outcomes. There is significant and growing interest among traditional development donors (such as DFID, USAID, and The World Bank), philanthropic institutions (such as UBS Optimus Foundation, Children’s Investment Fund Foundation, Bill & Melinda Gates Foundation, and Rockefeller Foundation), and the emerging class of impact investors, in the use of DIBs to effectively deliver impact in developing countries.^5^ One of the most advanced propositions currently in development is the area-wide control of zoonotic sleeping sickness in Uganda (34).

**Figure 2:**
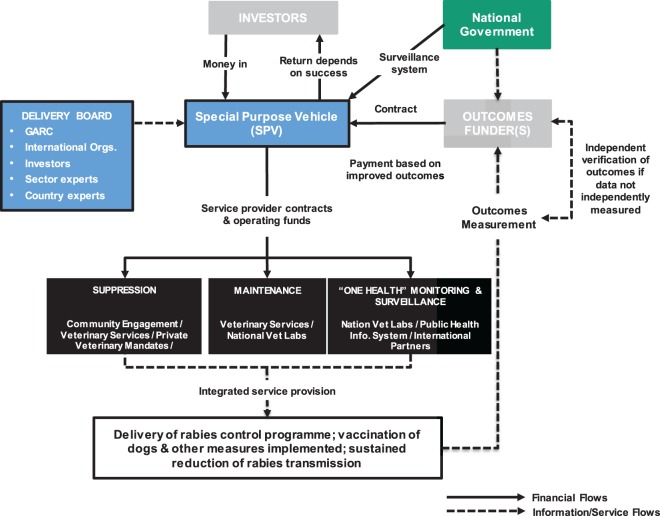
**Plausible Development Impact Bond (DIB) structure for rabies control**. The flow of money from the National Government to embed rabies surveillance post-vaccination control into the veterinary and public health systems may be through the SPV established to deliver the DIB or may be independent of the DIB structure but counts toward the overall delivery costs. Note, the structure was developed based on the work conducted with Global Alliance for Rabies Control (GARC) and is applicable for rabies control in general with other advocacy agencies playing similar roles to GARC.

Development Impact Bonds use private investment to provide up-front risk capital for development programs, only calling on donor (or government) funding to repay capital, plus a potential return (i.e., premium), once clearly defined and measured development outcomes are achieved. DIBs have the potential to attract new capital from impact investors motivated by both social and financial returns. By transferring the risk of program failure to these investors, DIBs bring a greater focus on implementation and delivery of successful results. In this way, DIBs also satisfy the growing demands that public funding, be it internal or spent on overseas aid, should be paid on successful results and in a transparent manner (Figure 2).

There are characteristics of infectious disease control programs in general, and rabies control in particular, which map neatly onto a DIB including

(1) *Strong evidence base that successful program delivery is technically achievable*.
  Development Impact Bonds are about scaling proven interventions and primarily look to shift implementation risk (not technical risk) from outcome payers to investors. The evidence base for effective rabies control has been validated and is strong. The constraints to implementation are known to be financial and operational. A consensus has emerged in the international community about the basis for implementing control with detail guidelines, *The Blueprint for Rabies Prevention and Control* (2014) developed by the Partners for Rabies Prevention (10), which includes the *Stepwise Approach to Rabies Elimination* tool (developed by FAO, the Global Alliance for Rabies Control, and other partners) and the approach endorsed by the WHO–OIE–FAO tripartite.^6^
  Models for designing and running effective rabies vaccination campaigns have been developed and trialed in a variety of developing country settings. For example, Tanzania, where rabies is endemic with an estimated 1,500 deaths each year (35), was among three countries selected by the WHO for large-scale rabies elimination demonstration trials between 2009 and 2013—funded by the Bill and Melinda Gates Foundation (BMGF) (see http://www.who.int/rabies/bmgf_who_project/en/index.html).
(2) *Substantial up-front investment is needed to unlock long-term net benefits*.
  The ideal cash-flow profile of the DIB (front loaded investment followed by long-term lower cash needs) mirrors the high up-front effort needed to interrupt rabies transmission followed by reduced effort to maintaining disease control. In poor countries, the money is rarely available to cover these up-front costs while traditional donor funding does not advance large amounts of cash; in the DIB, private investors provide the capital needed up front, at risk.
(3) *Affordable and sustainable maintenance of long-term impact*.
  Successful dog rabies mass vaccinations (providing sufficient coverage over sufficient time) will interrupt transmission allowing a shift to a disease-free maintenance phase based on surveillance, reactive vaccination, and appropriate human case management. Maintenance costs are significantly lower than the up-front costs of mass vaccination [e.g., Ref. (15, 36)], and there is the potential to embed the maintenance activities in routine public and veterinary health systems funded by the country government to ensure long-term sustainability beyond the end of the DIB.
(4) *Successful implementation requires coordination between multiple partners*.
  Controlling rabies requires engagement from partners across ministries and the private and public sector and demands a “One Health” approach in which there is close coordination between the veterinary and public health sectors. The investment structure in the DIB ensure a common drive to deliver specific outcomes providing a unified focus for the veterinary and human health delivery partners.
(5) *Tractable and affordable measure of outcomes that are valid indicators of long-term impact*.
  Tractable and affordable measure of outcomes will trigger payments from the outcome funders to the investors. For rabies, there exist established, validated, and robust diagnostic procedures to confirm positive rabies samples as well as case recording systems to monitor human rabies exposure. These measures provide a basis for quantifying the reduction in disease transmission relative to a pre-intervention baseline. The growing economic literature investigating the burden of rabies provides the evidence basis for understanding the impact unlocked by long-term reduction in rabies transmission.

## Structuring a DIB for Rabies Intervention

The structure of a DIB applied to rabies would partition interventions into four phases:

(1) *Pre-implementation phase* in which the detailed delivery plan is developed; baseline incidence data collected; existing public and veterinary health surveillance systems are, where necessary, strengthened and refined to provide the basis for tracking success across all phases of the intervention; reporting systems developed and tested; DIB infrastructure (e.g., establishment of special purpose vehicle for DIB contracting) and recruitment/contracting of delivery partners secured; payment triggers agreed; and an independent outcome auditor appointed.
(2) *Suppression phase* in which the mass vaccination campaigns are implemented at national level; routine reporting implemented; and audit of vaccination coverage by the independent outcome measurement group is conducted.
(3) *Consolidation phase* in which there is a shift from mass vaccination to surveillance and reactive vaccination following confirmed canine cases; protection of borders; audit of canine rabies incidence and suspect rabid dog bites.
(4) *Post-elimination maintenance phase* in which the surveillance capacity is embedded in government services and fully financed by the government.

For rabies, ideally DIB payment triggers would be split between a partial return of capital based on delivery of mass vaccination coverage (measured against the 70% target threshold) and a series of outcome payments (back-loaded to incentivize long-term sustainability) linked to a reduction in disease incidence in the reservoir dog population and also exposure in humans.

## Conclusion

The major constraint to progressing beyond a concept and launching a rabies-DIB is the lack of active engagement from a payer. Discussions with leading overseas development agencies, who have to act as the primary payers if DIBs are ever to be a significant source of financing in LMICs, have confirmed an interest among donors about DIBs in principle but in practice revealed a lack of internal expertise and capacity in engaging in the detailed planning of a DIB. This is not unexpected given the novelty of DIBs as a financing alternative to direct grant support. Moreover, novel structures are perceived as risky and so avoided. Part of the risk is the perception issues that a successful DIB will cost the payer more than direct grant funding. Another aspect of the risk is the lack of any large-scale working examples of a DIB, which itself is a function of the lack of donor backing to test a DIB and develop the evidence. To break this catch 22 situation the evidence generated from SIBs from developed settings should start to emerge to help support, or not, the theory of the impact bond financing approach. Despite increased advocacy for rabies, it should be noted that rabies is not perceived as a priority disease, even among the NTDs, and donors are positioning other development issues in the pipeline for possible DIB financing ahead of rabies.

Several approaches may help progress the DIB concept for NTD in general, and rabies more specifically, to accelerate the involvement of traditional government aid agencies:

First, an emerging theme in DIB design is the central importance of identifying an appropriate outcome measure and that can be tractably, affordably, and verifiably measured to provide a robust quantitative basis for triggering payments. This is central to all DIBs. These issues are complex for the NTDs, which are characterized by a high degree of underreporting in affected human populations and a particular problem for NZDs, where the work on sleeping sickness (37) and rabies (1) points to using measures of transmission in the animal reservoir population as a proxy measure for human disease burden. Potential locations suitable for pilot DIB-financed interventions are characterized by having an active, well-respected academic research group with a track-record of peer-reviewed papers detailing a robust understanding of the disease epidemiology and empirical evidence of successful pilot interventions; engaged local veterinary and human public health agencies and relevant central government support.

Second, although a single international donor may be reluctant to finance a DIB in full, there may be potential to attract a co-payer, such as a foundation or a national government. This would catalyze the involvement of donor agencies and stimulate the broader DIB market. In the case of rabies, the blueprint for rabies control can be used as a starting point to divide the task of global rabies elimination into a series of DIBs investments, scaled to investors. A philanthropic foundation that has previous been funding rabies control (e.g., The BMGF) could consider switching spend to cover part of a DIB payment. Similarly, a country that has previously benefited from donor funding for rabies control (e.g., South Africa where The BMGF has funded rabies vaccination through WHO) could undertake to cover part of the outcome payments and attract additional donors to secure the balance of outcome payment. A general DIB structure and site-specific example for rabies elimination in Chad have been developed and are currently market tested with investors and donors (36). The framework exists to develop other site-specific DIB proposal for rabies control, which local governments and non-governmental advocacy agencies can market to potential payers.

Finally, consideration should be given to how rabies control could be integrated into other NTD/NZD intervention platforms. While any one NTD/NZD may not be prioritized by a donor, an integrated approach that delivers multiple impacts through a common delivery platform could be attractive and highly cost effective. With a burden of 931,600 DALYs (38), the burden for rabies is higher that Chagas, cutaneous Leishmaniasis, trypanosomiasis, cysticercosis, echinococcisis, trachoma, yellow fever, Ebola, trichuriasis and leprosy (39), and programs could be aligned so adding value. For example, strengthening veterinary public health surveillance systems to track rabies cases could be beneficial for tracking the impact of interventions against several diseases, while awareness messaging could be extended to deliver important health messages against multiple disease in which dogs are important reservoirs of disease (including cutaneous Leishmaniasis and echinococcisis) (40–44). If additional interventions can be delivered at marginal costs utilizing the same delivery teams and infrastructure, then cost-effectiveness of each intervention is improved and the likelihood of donor support potentially increased. The integration of rabies into other large-scale intervention programs was an emerging theme at the recent Partners for Rabies Prevention meeting (May 2015). This could form the basis of a more compelling DIB, which developing country governments could prioritize and potentially unlock donor support.

## Author Contributions

All authors listed have made substantial, direct, and intellectual contribution to the work and approved it for publication.

## Conflict of Interest Statement

The authors declare that the research was conducted in the absence of any commercial or financial relationships that could be construed as a potential conflict of interest.

## Footnotes

^1^http://www.filariasis.org/.

^2^http://globalnetwork.org/.

^3^http://unitingtocombatntds.org/resource/london-declaration.

^4^http://www.who.int/neglected_diseases/NTD_RoadMap_2012_Fullversion.pdf.

^5^https://www.gov.uk/government/news/uk-development-bonds-will-combat-global-poverty.

^6^http://www.rabiesblueprint.com/.
